# Opium use, cigarette smoking, and alcohol consumption in relation to pancreatic cancer

**DOI:** 10.1097/MD.0000000000003922

**Published:** 2016-07-18

**Authors:** Ramin Shakeri, Farin Kamangar, Mehdi Mohamadnejad, Reza Tabrizi, Farhad Zamani, Ashraf Mohamadkhani, Sepideh Nikfam, Arash Nikmanesh, Masoud Sotoudeh, Rasoul Sotoudehmanesh, Bijan Shahbazkhani, Mohammad Reza Ostovaneh, Farhad Islami, Hossein Poustchi, Paolo Boffetta, Reza Malekzadeh, Akram Pourshams

**Affiliations:** a Digestive Oncology Research Center, Digestive Diseases Research Institute, Tehran University of Medical Sciences, Tehran, Iran; b Digestive Disease Research Center, Digestive Diseases Research Institute, Tehran University of Medical Sciences, Tehran, Iran; c Department of Public Health Analysis, School of Community Health and Policy, Morgan State University, Baltimore, MD; d Liver and Pancreatobiliary Diseases Research Center, Digestive Diseases Research Institute, Tehran University of Medical Sciences; e Gastrointestinal and Liver Disease Research Center, Firoozgar Hospital, Iran University of Medical Sciences; f Sasan Alborz Biomedical Research Center, Masoud Gastroenterology and Hepatology Clinic, Tehran, Iran; g Division of Gastroenterology and Hepatology, The Sol Goldman Pancreatic Cancer Research Center, The Johns Hopkins Medical Institutions, Baltimore, MD; h American Cancer Society, Atlanta, GA; i Institute for Transitional Epidemiology and the Tisch Cancer Institute, Mount Sinai School of Medicine, New York, NY.

**Keywords:** alcohol, opium, pancreatic cancer, tobacco

## Abstract

**Background and Aims:**

Although several studies have suggested opium as a risk factor for cancers of the esophagus, stomach, larynx, lung, and bladder, no previous study has examined the association of opium with pancreatic cancer. We aimed to study the association between opium use and risk of pancreatic cancer in Iran, using a case-control design. We also studied the association of cigarette smoking and alcohol consumption with pancreatic cancer, for which little information was available from this population.

**Methods:**

Cases and controls were selected from patients who were referred to 4 endoscopic ultrasound centers in Tehran, Iran. We recruited 316 histopathologically (all adenocarcinoma) and 41 clinically diagnosed incident cases of pancreatic cancer, as well as 328 controls from those with a normal pancreas in enodosonography from January 2011 to January 2015. We used logistic regression models to calculate odds ratios (ORs) and 95% confidence intervals (CIs).

**Results:**

After adjustment for potential confounders, opium use (OR 1.91; 95% CI 1.06–3.43) and alcohol consumption (OR 4.16; 95% CI 1.86–9.31) were significantly associated with an increased risk of pancreatic cancer. We did not find an association between ever tobacco smoking and pancreatic cancer risk (OR 0.93; 95% CI 0.62–1.39).

**Conclusion:**

In our study, opium use and alcohol consumption were associated with an increased risk of pancreatic cancer, whereas cigarette smoking was not.

## Abstract 2

Supplemental Digital Content is available in the text

## 1 Introduction

According to the United Nations Office of Drugs and Crime (UNODC), an estimated 16.5 million people use opium or its derivatives illicitly.^[1]^ The acute effects of opium use—such as its analgesic and soporific effects—have been known for millennia. However, its long-term effects on health, such as its potential carcinogenic effects, have been studied only since the 1970s, mostly over the past decade.^[2]^ Several case-control and cohort studies have suggested that opium use may increase the risk of cancers of the esophagus,^[3–6]^ stomach,^[4,7–9]^ larynx,^[10,11]^ lung,^[12–14]^, and bladder.^[15–21]^ These epidemiologic findings are supported by laboratory studies showing that, after metabolic activation, opium dross has mutagenic activity in *Salmonella typhimurium* strains TA98 and TA100;^[22]^ causes frameshift mutations in *S typhimurium* strains TA1538 and TA98;^[23]^ and induces sister chromatid exchanges.^[24]^ These effects are mostly attributed to nitrogen-containing heterocyclic compound derived from pyrolysis of morphine.^[25]^ To our knowledge, no previous study has examined its association with pancreatic cancer.

Cigarette smoking is a known cause of pancreatic cancer. Several meta-analyses have shown increased risk of pancreatic cancer as a result of cigarette smoking, mostly with odds ratios (OR) of 1.5 to 2.0, depending on the duration and intensity of smoking.^[26–28]^ However, the association of smoking with various cancers in Iran has often been much less strong than what is seen in Western countries.^[5,9,14,29]^ Alcohol consumption may be another risk factor; whereas some studies have shown no increased risk, others have suggested that alcohol consumption may be associated with higher risk of pancreatic cancer. A recent meta-analysis indicated that heavy, but not low consumption of alcohol, was associated with an increased risk of pancreatic cancer.^[30]^

We aimed to study the association between opium use and risk of pancreatic cancer in Iran, using a case-control design. We have also investigated the association between cigarette smoking and alcohol consumption with risk of pancreatic cancer in our study population.

## 2 Methods

### 2.1 Overall design and study procedures

This case-control study was approved by the Institutional Review Board of Digestive Disease Research Center, Tehran University of Medical Sciences (IRB number: IRB00001641, Federal wide Assurance number: FWA00015916). Study participants were recruited from patients referred for endoscopic ultrasonography (EUS) to 3 tertiary referral hospitals (Shariati, Firoozgar, and Atieh Hospitals) or a specialty clinic (Masoud Clinic) in Tehran, Iran, from January 2011 to January 2015. Patients were visited and assessed clinically by an endosonographist for one of the following reasons: suspicion for a mass or cyst in the pancreas or bile ducts; assessment of submucosal lesions found during esophago-gastro-duodenal endoscopy; or to rule out bile duct stones. If, based on history and clinical assessment, the endosonogrphist had a suspicion of pancreatic mass, the patient was asked to participate in the study. This invitation was made based on suspicion only and before endosonography was done. After obtaining informed consent, the patient was referred to a trained general practitioner for interview and biological sample collection. Potential study participants were administered a detailed questionnaire; provided blood, saliva, and urine samples; and underwent EUS. The tissue samples obtained during EUS were read by expert pathologists. Cases (those with pancreatic adenocarcinoma) and controls (those without pancreatic adenocarcinoma) were selected based on the results of history of their signs and symptoms, clinical exams, EUS, histopathology, and other information obtained during data collections.

### 2.2 Questionnaire data

Trained general practitioners administered a structured questionnaire with 113 questions to each study participant. The questionnaire was completed before endosonography was done for 2 reasons: (1) the physicians and the patients were unaware of the final diagnosis; therefore, the chance of interviewer or responder bias was reduced; and (2) the patients could respond to questions more accurately before receiving sedation for endosonography. We collected data on demographics; anthropometric indices; socioeconomic status indicators; signs and symptoms of the current disease; occupational history and exposure to certain physical and chemical agents; medical and drug history; family history of cancer; history of alcohol, tobacco, or opium use; history of tea and coffee consumption; pregnancy and menstrual data (only for women); and dietary habits and cooking methods. This questionnaire was tested for and showed excellent validity and reliability.^[31]^

Questions on opium use included being an ever user (having used at least weekly for a period of 6 months or more), age of initiation of use, duration of use (in years), frequency of use (per week), and typical amount of use (in a local unit called nokhod, roughly 0.2 g). Questions also included routes of use (ingestion, inhalation, and injection) and types of opium locally used (teriak, shireh, sukhteh, and heroin). Teriak is the air-dried sticky paste of raw opium. Sukhteh is the dry, black residue of smoked teriak, which sticks to the opium pipe. It can be scraped from the opium pipe and ingested. Shireh is refined opium product often made by boiling a combination of raw opium and sukhteh in hot water and passing the solution through filters several times.

### 2.3 Biological sample collection and other assessments

The general practitioners also measured height and weight of the patients and collected samples of blood, saliva, and urine using predefined protocols. All biological samples were transferred to –20 ^o^C freezers within 15 minutes of collection, and then to –70 ^o^C freezers within 48 hours. The practitioners also collected a copy of all previous patient records, including medical charts, laboratory data, and diagnostic imaging in a file.

*EUS:* All of the above patients were offered EUS, and if they had mass or cystic lesions, underwent fine needle aspiration (FNA). The only exception was those who were highly suspicious for insulinoma based on laboratory findings. One of four expert endosonographists, each with >5 years of experience, conducted the exams. The endosonographists attempted at least 3 needle passes to obtain adequate tissue. If adequate tissue was not obtained using this method, histological sampling was done using a second fine needle aspiration, or rarely under the computed tomography (CT) scanning. Pentax EUS machines were used in Shariati and Atieh hospitals and Masoud Clinic, whereas a Fujinon machine was used in Firoozgar Hospital.

### 2.4 Histopathological exam

An experienced pathologist evaluated all samples collected using EUS or other methods described above. If a definitive diagnosis of ductal adenocarcinoma could not be made using hematoxylin/eosin (H&E) staining, the samples were evaluated using an immunohistochemistry panel to differentiate ductal adenocarcinoma from neuroendocrine tumors, pseudopapillary tumors, or lymphoma.

*Cases:* For this study, pancreatic cancer cases defined as those who either received a histopathological diagnosis of pancreatic adenocarcinoma or were clinically diagnosed as pancreatic cancer.

### 2.5 Controls

Controls were those who had all of the following qualifications: normal pancreas in the EUS exam; age 40 years or older; a final diagnosis of either asymptomatic small (<10 mm) submucosal lesion in the esophagus or stomach, or a gallbladder or common bile duct stones without cholangitis; no history or current diagnosis of liver failure or renal failure; no history of cancer; no adherence to special diets; no diagnosis of opium-induced common bile duct dilatation or sphincter of Oddi dysfunction; and no development of pancreatic disease or any cancers 1 year after the initial visit. There is substantial literature ^[32–36]^ showing that opium could cause common bile duct dilatation or sphincter of Oddi dysfunction. However, such diagnosis was made only after careful investigation and ruling out all other causes. This last criterion (no cancer after 1 year of follow up) was applied to make sure that there no subclinical lesions or was no misdiagnosis in the initial visit. One year after the initial visit, the general practitioner had a phone conversation with the patient or their relative to ensure that no pancreatic disease or other disease had developed during that year. Patients who did not match one or more of the above-mentioned criteria (e.g., those with submucosal lesions of >10 mm) were excluded from the list of controls.

### 2.6 Statistical analysis

Statistical analyses were conducted with Stata statistical software, version 11 (STATA Corp, College Station, TX). Frequencies and percentages were calculated for categorical variables, and means and standard deviations were calculated for continuous variables. We used logistic regression models to calculate odds ratios (OR) and 95% confidence intervals (95% CI) for the association of the main exposures (opium use, cigarette smoking, and alcohol consumption) with case status. In addition to unadjusted results, we present the ORs and 95% CIs adjusted for potential confounders, including age, sex, place of residence (urban or rural), and mutual consumption of opium use, smoking, and alcohol consumption. Education and marital status were not associated with pancreatic cancer risk and therefore were not included in the final models.

In addition to analyzing the main exposures as dichotomous variables (ever vs never), we analyzed and show the association for average amount of use, duration of use, and cumulative use (defined as duration of use, in days, multiplied by daily amount of use). We show OR (95% CI) for the association of opium use by routes of administration.

A main concern for the association of opium and case status is reverse causality, as patients may use opium to alleviate pain. To address reverse causality, we also dropped from analysis any opium use during 1, 2, and 3 years prior to diagnosis.

## 3 Results

Of the initial pool of 1726 patients who underwent EUS from January 2011 to January 2015, a total of 357 cases of pancreatic cancer and 328 controls were enrolled in this study.

Table 1 compares demographic characteristics and other potential confounders of interest for cases and controls. The mean (standard deviation) age for controls and cases were 64.6 (11.5) versus 64.7 (11.7) years, respectively; 45.4% of controls and 60.5% of the cases were males. Controls and cases were substantially different in terms of place of residence (urban vs rural) but not for ethnicity, marital status, or education.

**Table 1:**
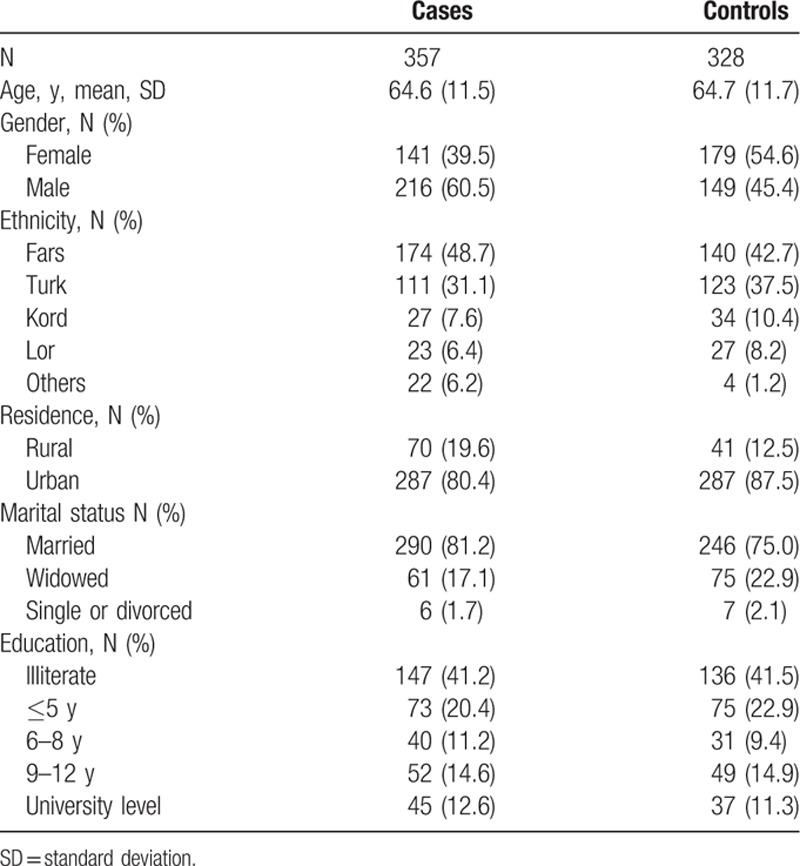
Demographic characteristics of pancreatic cancer cases and controls.

Table 2 shows adjusted and unadjusted results for the association between opium use and pancreatic cancer. Overall, 57 cases (16.0%) compared to only 21 controls (6.4%) had ever used opium, resulting in an unadjusted OR (95% CI) of 2.77 (1.64–4.69) and an adjusted OR (95% CI) of 1.91 (1.06–3.43). To ensure that opium use was not started because of cancer-related pain, in a sensitivity analysis we reclassified the cancer cases who started opium use within 1, 2, and 3 years prior to diagnosis as nonusers. This reclassification led to the exclusion of 2, 3, and 5 exposed cases, respectively, and no exposed controls. The adjusted OR (95% CI) was of 1.82 (1.01–3.29) for reclassifying the results for 1 year prior to diagnosis, which was statistically significant. After reclassifying the results for 2 or 3 years prior to diagnosis, the adjusted OR (95% CI) were 1.76 (0.97–3.18) and 1.69 (0.93–3.05), respectively. Although these adjusted ORs were not significant, the ORs were still substantially above 1 and very close to the original results.

**Table 2:**
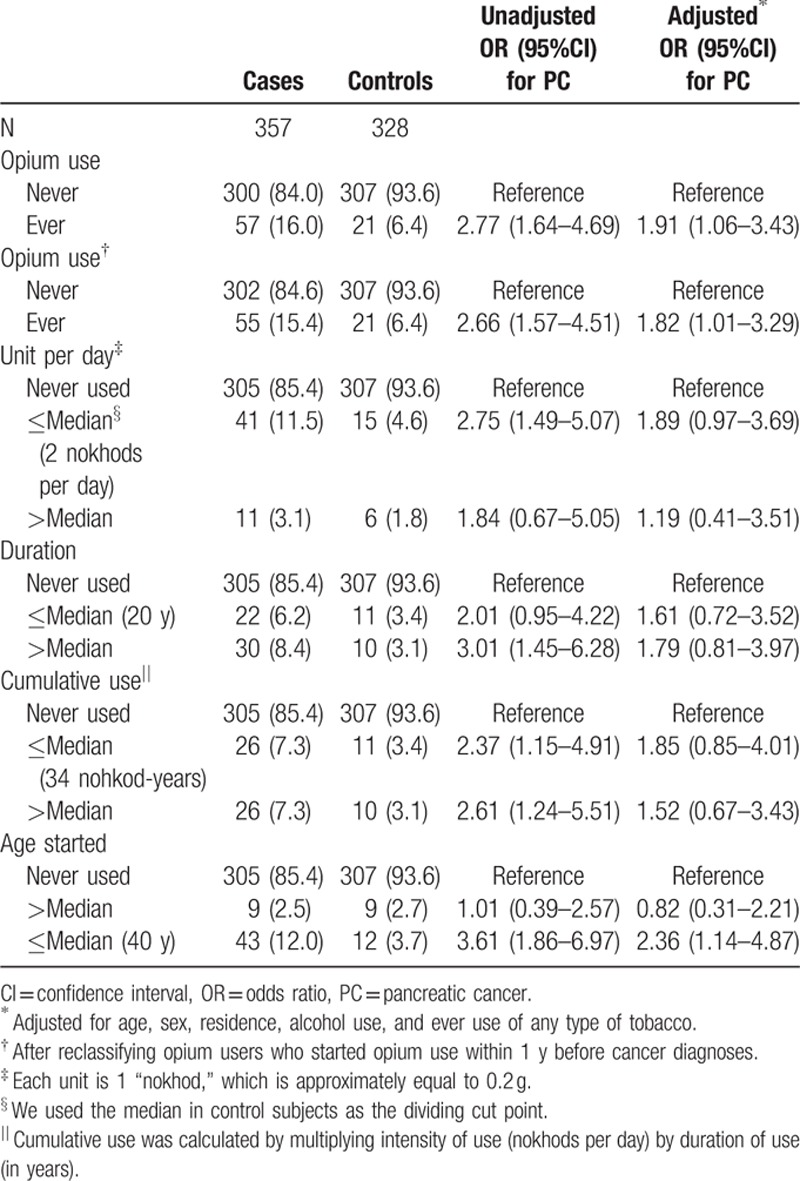
Opium use in pancreatic cancer cases and controls.

Further analyses (Table 2) did not indicate a dose–response relationship between opium use and pancreatic cancer. No higher risk was associated higher average intensity of dose, longer duration of use, or cumulative use.

Table 3 demonstrates the results for association of cigarette smoking with pancreatic cancer. Whereas the unadjusted results show a statistically significant association (OR 1.52; 95% CI 1.09–2.12), the adjusted results show almost no association (OR 0.93; 95% CI 0.62–1.39). Further analyses indicated no evidence for a dose–response association. The main confounding factors were sex, opium use, and alcohol consumption.

**Table 3:**
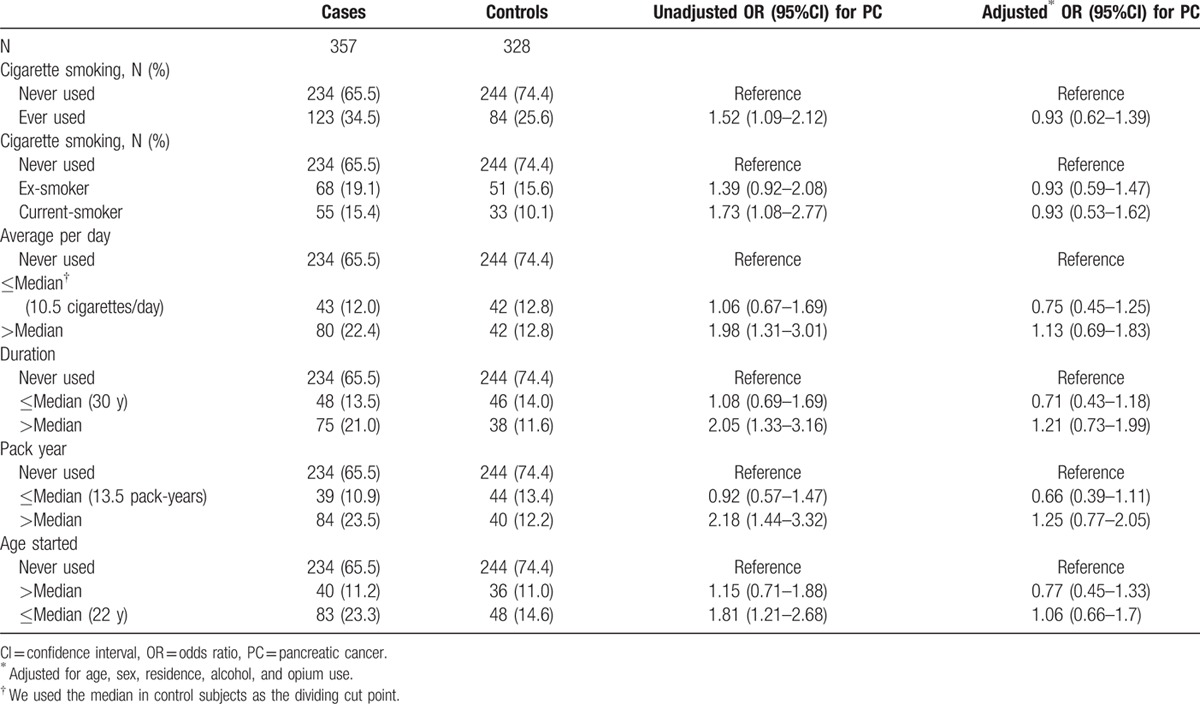
Cigarette in pancreatic cancer cases and controls.

The association between alcohol consumption and pancreatic cancer is shown in Table 4. Among cases, 39 (10.9%) had consumed alcohol, compared to 8 controls (2.4%), resulting in an unadjusted OR (95%) CI of 5.33 (2.46–11.54) and an adjusted OR (95% CI) of 4.16 (1.86–9.3). Longer duration of use, but not higher cumulative use, was associated with a higher risk.

**Table 4:**
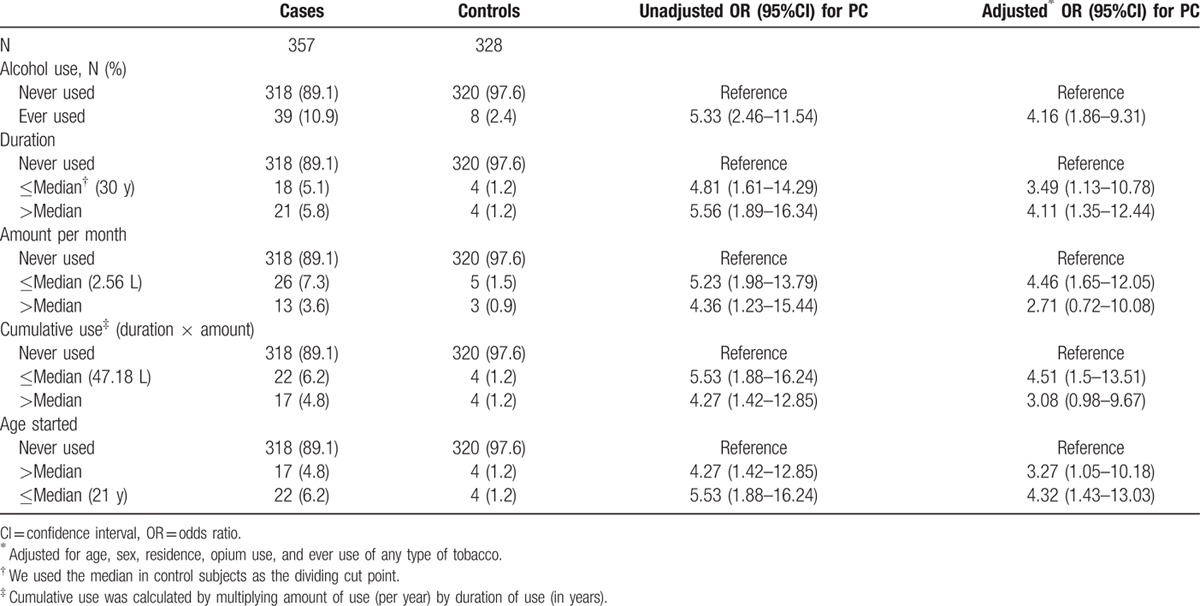
Alcohol use in pancreatic cancer cases and controls.

Finally, we have illustrated the relationship of pancreatic cancer and alcohol, opium, and tobacco use with weighted markers, in supplementary Figure 1.

## 4 Discussion

In our study population, opium use and alcohol consumption, but not cigarette smoking, were associated with an increased risk of pancreatic cancer.

Whereas this is the first study of opium in relation to pancreatic cancer, the results support the currently existing literature indicating that opium use may be carcinogenic. Several methodologic problems need to be considered before making a causal conclusion. The association between opium use and higher risk of cancer may be confounded by other risk factors, such as age, sex, tobacco use, and alcohol use. Opium users are more likely to be older men who also smoke cigarettes.^[37]^ However, adjustment for these potential confounders did not substantially change the results and the associations remained statistically significant.

Reverse causality is another concern, as people who have early forms of cancer may use opium to alleviate their pain. To address this concern, we conducted a sensitivity analyses by reassigning cancer cases who initiated opium use within 1 year prior to diagnosis as nonusers. With this reclassification, the odds ratios were reduced but were still statistically significant. After reclassifying the cases who started opium use 2 or 3 years prior to diagnosis, the results were no longer statistically significant but the odds ratios remained much >1 and close to the original values. However, the rate of early diagnosis of pancreas cancer is very low ^[38]^ and by the time a person has symptoms, the cancer is in its late stages and tumor is significantly large. On the other hand, pancreatic cancer is a fast-progressing cancer, after it becomes detectable, ^[39]^ and rarely produces symptoms several years prior to cancer, so we believe that excluding cases who only used opium 1 or 2 years prior to diagnosis is acceptable timeframe to address the concern of reverse causality.

Other issues of concern are biases that may arise from a case-control design, including recall bias and selection bias. Recall bias is unlikely, as there is little literature on opium and pancreatic cancer to bias the opinion of the cases. More importantly, cases and controls reported their opium use prior to the diagnosis. Therefore, their answers could have not been biased. However, selection bias is possible, in ways that may both accentuate or reduce the odds ratios. For comparability, controls were selected from among those who were referred for EUS, which included patients with common bile duct stones. If such control subjects used opium to alleviate pain, the results may be biased toward null. In contrast, patients with the diagnosis of opium-induced bile duct dilatation were excluded from the pool of controls, which could bias the results away from null. Prospective studies offer an advantage to avoid these potential shortcomings.

The finding that pyrolysates of morphine, the most abundant alkaloid of opium, shows strong mutagenic effects, and a discovery of a new class of very strong class of mutagens in these pyrolystaes,^[25]^ lends support to the hypothesis that opium could cause pancreatic cancer. However, the lack of a dose–response association in our findings reduces the support for causality.

In our study population, after adjustment for other factors, cigarette smoking was not associated with a higher risk of pancreatic cancer. Whereas this finding is different from many studies published across the world and resulting meta-analyses,^[26–28]^ it is consistent with many other studies of smoking and cancer in Iran and some other Asian countries.^[29,40]^ The association of cigarette smoking with risk of several cancers, including cancers of the esophagus,^[5]^ stomach,^[9]^ lung,^[41]^ all cancers combined,^[14]^ and total mortality,^[14]^ is far less strong than what is seen in Western countries. The reasons for this pattern are unclear, although this could be related to differences in the intensity of smoking or other smoking behaviors in Western and most Asian countries,^[42,43]^ further investigation of this phenomenon is worthwhile.

In this study, alcohol consumption was associated with a substantial increase in risk of pancreatic cancer. Alcohol consumption is a strong risk factor for pancreatitis.^[44,45]^ Meta-analyses have found an association between high consumption, not low consumption, of alcohol with risk of pancreatic cancer.^[30]^ Our study shows one of the strongest associations found between alcohol and pancreatic cancer in the literature. We are not sure whether this strong association is causal due to alcohol itself, or it is a byproduct of the types of alcohol used in Iran, or it is simply a matter of confounding. Alcohol use is illegal in Iran, therefore, much of the alcohol used in Iran over the past 35 years has been home-made. Due to its illegal nature, there is no overseeing of production, and adulterants may be used. Whereas we have adjusted for several factors, residual confounding is still possible, as alcohol consumers in Iran may be different from the rest of the population in ways that we cannot exactly pinpoint.

This study has some strengths and limitations. This article reports the first epidemiologic study of opium use in relation to pancreatic cancer. Relatively large sample size, detailed questions on opium use and potential confounders, uniform data collection, selecting cases and controls from the same clinics, questioning the patients for opium use prior to diagnosis, and a strict case and control selection criteria are also among the strengths. The main limitation is that of most case-control studies of cancer, that is, a potential for selection bias.

In conclusion, this study suggests that opium use and alcohol consumption may be risk factors for pancreatic cancer in Iran. The results for opium use are consistent with previous literature showing that opium is associated with a host of other cancers. Further studies, particularly prospective studies, may shed further light on this association.

## Supplementary Material

Supplemental Digital Content
