# Supplementary figures and images for: Opium use, cigarette smoking, and alcohol consumption in relation to pancreatic cancer
Source: Medicine (Baltimore). 2016 Jul 18;95(28):e3922. doi: 10.1097/MD.0000000000003922 (PMC4956779; doi:10.1097/MD.0000000000003922)

Supplementary Figure1: Relationship of pancreatic cancer and alcohol, opium, and tobacco use


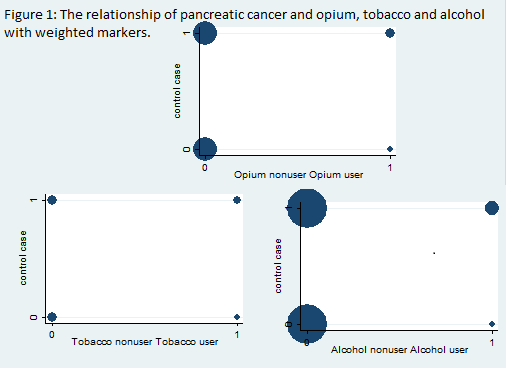

Supplement: Supplemental Digital Content [file medi-95-e3922-s001.doc]
